# ENOblock, a unique small molecule inhibitor of the non-glycolytic functions of enolase, alleviates the symptoms of type 2 diabetes

**DOI:** 10.1038/srep44186

**Published:** 2017-03-08

**Authors:** Haaglim Cho, JungIn Um, Ji-Hyung Lee, Woong-Hee Kim, Wan Seok Kang, So Hun Kim, Hyung-Ho Ha, Yong-Chul Kim, Young-Keun Ahn, Da-Woon Jung, Darren R. Williams

**Affiliations:** 1 New Drug Targets Laboratory, School of Life Sciences, Gwangju Institute of Science and Technology, 1 Oryong-Dong, Buk-Gu, Gwangju, 61005, Republic of Korea; 2 Cell Regeneration Research Center, Department of Cardiology, Cardiovascular Center, Chonnam National University Hospital, 671 Jebong-ro, Dong-gu, Gwangju, 501-757, Korea; 3 Division of Endocrinology and Metabolism, Inha University School of Medicine, 400-711, Republic of Korea; 4 College of Pharmacy and Research Institute of Life and Pharmaceutical Sciences, Sunchon National University, Sunchon, 540950, Republic of Korea; 5 Drug Discovery Laboratory, School of Life Sciences, Gwangju Institute of Science and Technology, 1 Oryong-Dong, Buk-Gu, Gwangju, 61005, Republic of Korea

## Abstract

Type 2 diabetes mellitus (T2DM) significantly impacts on human health and patient numbers are predicted to rise. Discovering novel drugs and targets for treating T2DM is a research priority. In this study, we investigated targeting of the glycolysis enzyme, enolase, using the small molecule ENOblock, which binds enolase and modulates its non-glycolytic ‘moonlighting’ functions. In insulin-responsive cells ENOblock induced enolase nuclear translocation, where this enzyme acts as a transcriptional repressor. In a mammalian model of T2DM, ENOblock treatment reduced hyperglycemia and hyperlipidemia. Liver and kidney tissue of ENOblock-treated mice showed down-regulation of known enolase target genes and reduced enolase enzyme activity. Indicators of secondary diabetic complications, such as tissue apoptosis, inflammatory markers and fibrosis were inhibited by ENOblock treatment. Compared to the well-characterized anti-diabetes drug, rosiglitazone, ENOblock produced greater beneficial effects on lipid homeostasis, fibrosis, inflammatory markers, nephrotoxicity and cardiac hypertrophy. ENOblock treatment was associated with the down-regulation of phosphoenolpyruvate carboxykinase and sterol regulatory element-binding protein-1, which are known to produce anti-diabetic effects. In summary, these findings indicate that ENOblock has potential for therapeutic development to treat T2DM. Previously considered as a ‘boring’ housekeeping gene, these results also implicate enolase as a novel drug target for T2DM.

Type 2 diabetes mellitus (T2DM) accounts for approximately 90% of all cases of diabetes and has a major impact on human health and society^1^. Moreover, the global prevalence of T2DM is increasing to reach epidemic proportions^2^. T2DM is linked with obesity and the development of significant comorbidities, such as liver, heart and kidney disorders^3^. Therefore, the management of T2DM includes strategies to treat hyperglycemia and obesity, as well as the prevention of comorbidities. Currently available anti-diabetes drugs have limited efficacy, may fail to treat obesity or the development of secondary complications, and/or may have concerns about side effects^4^. For example, the commonly prescribed anti-diabetes drug, rosiglitazone, was subsequently found to produce significant cardiac-related events^5^. Therefore, the identification of new candidate agents and drug targets with the potential to treat diabetes and its secondary complications is a research priority.

Glycolysis is an ancient, highly conserved catabolic pathway of 10 biochemical reactions that converts glucose into pyruvate. This pathway generates the high energy-containing compounds adenosine triphosphate (ATP) and reduced nicotinamide adenine dinucleotide (NADH). Glycolysis enzymes are known to have additional, non-glycolytic roles in cellular physiology, which has been termed ‘moonlighting.’^6^ Small molecule screening and target validation has shown that two glycolysis enzymes, glyceraldehyde 3-phosphate dehydrogenase (GAPDH) and enolase, possess moonlighting functions that can produce potential anti-diabetic effects^7^,^8^,^9^. Recently, the novel enolase modulating compound, ENOblock, was shown to induce two potentially anti-diabetic effects: increased glucose uptake and down-regulation of the gluconeogenesis enzyme, phosphoenolpyruvate carboxykinase (Pck-1) in cells and wild-type zebrafish larvae^9^,^10^. However, the anti-diabetic effects of enolase modulation by ENOblock in mammals has not been tested.

In this study, we investigated the potential of ENOblock to modulate enolase moonlighting and produce therapeutic effects in a mammalian model of T2DM. ENOblock was compared with the known anti-diabetes drug, rosiglitazone^11^. We observed that ENOblock treatment reduces enolase activity *in vivo*, induces the nuclear translocation of enolase and produces anti-diabetic, anti-inflammatory and anti-fibrotic effects. Compared to rosiglitazone, ENOblock treatment produced less adverse side effects in the liver, kidney and heart. These findings implicate enolase as a novel drug target for T2DM and ENOblock as a T2DM drug candidate for clinical development.

## Results

### ENOblock induces enolase nuclear localization and modulates target gene expression

**Figure 1:**
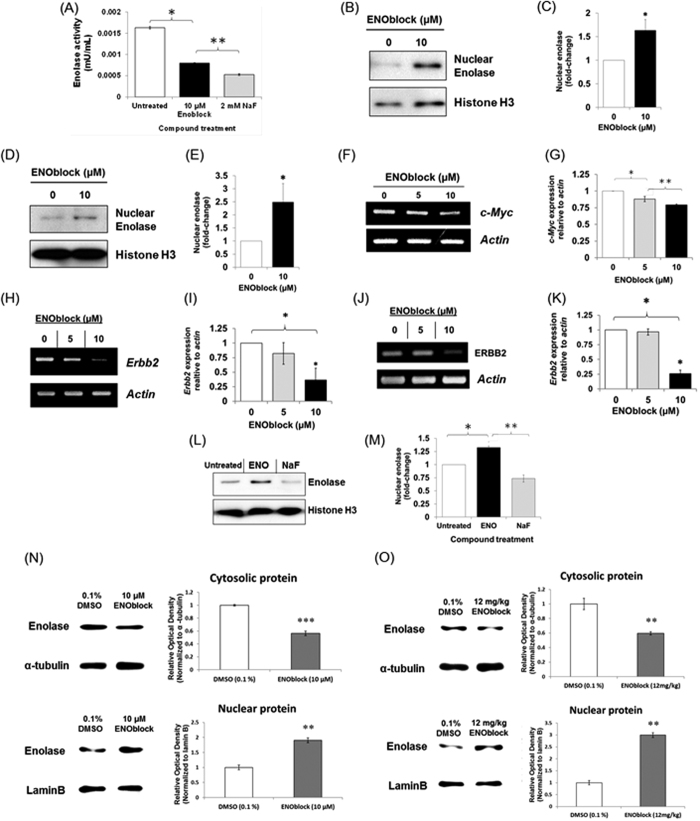
(**A**) Treatment of NIH/3T3 fibroblasts with 10 μM ENOblock or 2 mM NaF (a known enolase enzyme inhibitor) for 48 h decreased enolase activity. (**B**) Western blot analysis of enolase nuclear localization in 3T3-L1 pre-adipocytes treated with 10 μM ENOblock for 48 h. (**C**) Quantification of enolase nuclear localization. (**D**) Western blot analysis of enolase nuclear localization in Huh7 hepatocytes treated with 10 μM ENOblock for 48 h. (**E**) Quantification of enolase nuclear localization. (**F**) RT-PCR analysis of the known enolase-binding gene, c-Myc expression in 3T3-L1 pre-adipocyte cells treated with ENOblock for 48 h. (**G**) Real-time PCR quantification of c-Myc expression in 3T3-L1 pre-adipocyte cells treated with ENOblock. (**H**) RT-PCR analysis of the enolase-binding gene, Erbb2 expression in 3T3-L1 pre-adipocyte cells treated with ENOblock for 48 h. (**I**) Real-time PCR quantification Erbb2 expression. (**J**) RT-PCR analysis of the known enolase-binding gene, Erbb2 expression in Huh7 hepatocytes treated with ENOblock for 48 h. (**K**) Real-time PCR quantification of Erbb2 expression in Huh7 cells treated with ENOblock. (**L**) Western blot to show nuclear localization of enolase in Huh7 hepatocytes treated with 10 μM ENOblock or 2 mM NaF (an enolase catalytic site inhibitor) for 48 h. (**M**) Quantification of enolase nuclear localization. (**N**) Cytoplasmic and nuclear enolase protein expression in mouse primary hepatocytes after treatment with 10 μM ENOblock for 72 h. Cytoplasmic enolase was normalized with α-tubulin and nuclear enolase was normalized with lamin B. (**O**) Cytoplasmic and nucleic enolase expression in C57BL/6 J mouse liver tissue after treatment with 12 mg/kg ENOblock for 24 h. Cytoplasmic enolase was normalized with α-tubulin and nuclear enolase was normalized with lamin B. Statistical analysis: for (**A**–**M**): *p < 0.01 compared to untreated; **p < 0.01 compared to ENOblock treated; ***p < 0.001 compared to 0.1% DMSO treated (Student’s *t* test). Error = SEM for (**A**); Error = SD for (**B**–**M**); n = 3. For (**N**–**O**): Statistical analysis was carried out with a one-way ANOVA test. *ns*: not significantly different. *, ** or ***: significantly different from the corresponding control (DMSO (0.1%) - treated) respectively with *p* < 0.05, *p* < 0.01 or *p* < 0.001; Error = SEM. Western blots and RT-PCR gels are representative images from the treated mice or cells.

The ENOblock compound was developed as a probe to study the non-glycolytic functions of enolase^9^. To assess if this compound also reduces enolase enzyme activity, fibroblasts were treated with ENOblock. It was observed that ENOblock treatment reduced enolase activity in the cells (Fig. 1A). Enolase and its nuclear isoform, Myc-binding protein-1 (MBP-1), can bind DNA and act as a transcriptional repressor^12^,^13^,^14^. Therefore, we measured the nuclear localization of enolase after ENOblock treatment. It was observed that treating 3T3-L1 pre-adipocytes or Huh7 hepatocytes with ENOblock increased the level of enolase in the nucleus (Fig. 1B–E). ENOblock treatment decreased expression of c-Myc in adipocytes, which is a known target gene for nuclear enolase/MBP-1 (Fig. 1F,G)^15^. Erbb2 (HER2/neu), which is also a known enolase target gene^16^, showed decreased expression after ENOblock treatment in both hepatocytes and adipocytes (Fig. 1H–K). To assess if ENOblock induces enolase nuclear localization via modulation of enzyme glycolytic activity, hepatocytes were treated with NaF, which inhibits enolase catalytic activity. NaF did not induce nuclear localization, indicating non-glycolytic modulation of enolase by ENOblock to increase nuclear localization (Fig. 1L–M). To confirm that the protein purification protocol separates nuclear and cytoplasmic proteins, α-tubulin and laminB were also used as a cytoplasmic and nuclear marker, respectively (Supplementary Figure 1). The ability of ENOblock to induce nuclear translocation of enolase was also demonstrated using primary murine hepatocytes (Fig. 1N) and in the liver tissue of mice treated with ENOblock (Fig. 1O).

### ENOblock reduces blood glucose, LDL cholesterol and enolase activity in T2DM mice

**Figure 2:**
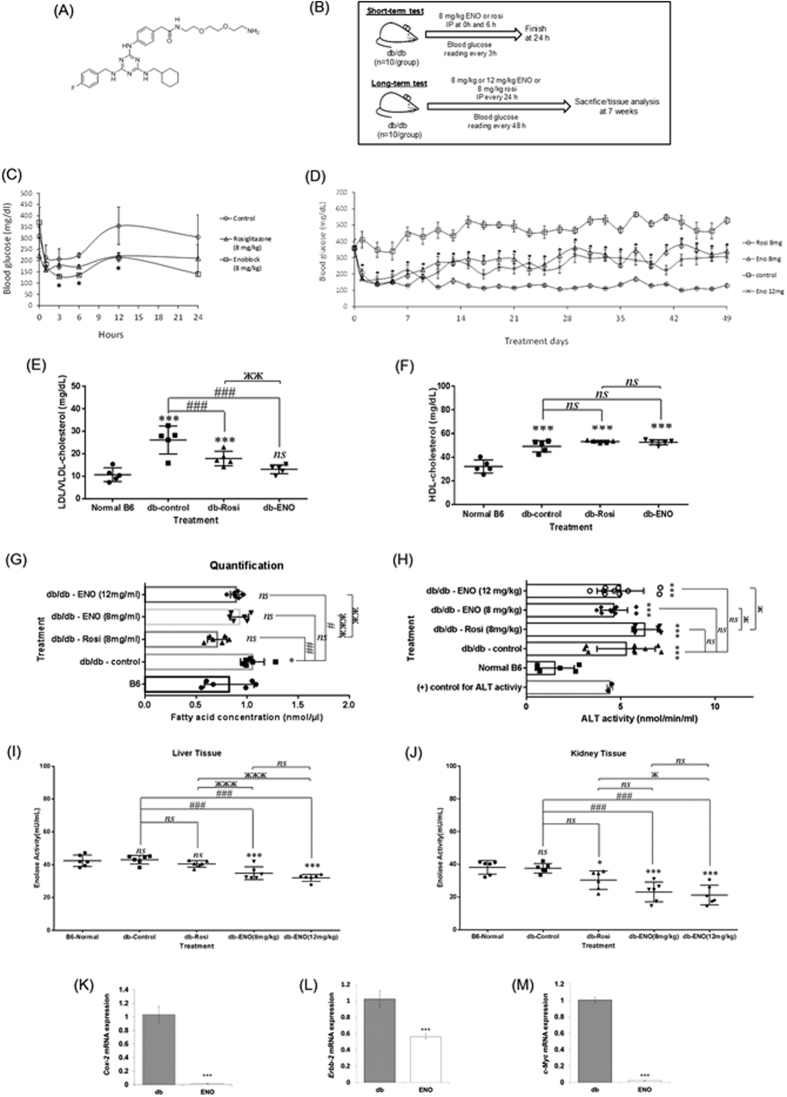
(**A**) Chemical structure of ENOblock (molecular weight = 631.18). (**B**) Schematic of the protocols to assess the anti-diabetic effects of ENOblock in db/db T2DM mice. (**C**) Blood glucose level for 24 h in db/db mice treated with one dose of 8 mg/kg ENOblock or 8 mg/kg rosiglitazone. **(D**) Blood glucose level in db/db mice treated over 7 weeks with 8 mg/kg or 12 mg/kg ENOblock or 8 mg/kg rosiglitazone. (**E**) LDL cholesterol level in db/db mice after 7 weeks treatment with 12 mg/kg ENOblock or 8 mg/kg rosiglitazone. (**F**) HDL cholesterol in db/db mice after 7 weeks treatment with 12 mg/kg ENOblock or 8 mg/kg rosiglitazone. (**G**) The free-fatty acid level in the serum of the treated mice. (**H**) ALT activity in the serum of the treated mice. (**I**) Enolase activity in the liver tissue of treated mice. (**J**) Enolase activity in the kidney tissue of treated mice. For (**E**–**J**), age-matched, background strain BL6 mice were used for comparison. (**K**–**M**) Real-time PCR analysis of the known enolase target genes Cox-2, c-Myc and Erbb-2 in liver tissue of db/db mice treated with 12 mg/kg ENOblock for 7 weeks. Statistical analysis for Fig. 2 (**C**,**D**) and (**K**–**M**) statistical analysis was carried out using the Student’s *t* test. Error = SEM. *p < 0.05 for ENOblock 8 mg/kg compared untreated db/db mice. (**E**–**J**) statistical analysis was carried out with a one-way ANOVA test, followed by a Dunnett’s mutiple comparisons test and unpaired *t* - test. *ns*: not significantly different. *, ** or ***: significantly different from the corresponding normal B6 respectively with *p* < 0.05, *p* < 0.01, *p* < 0.001; #, ## or ###: significantly different from the corresponding db-control respectively with *p* < 0.05, *p* < 0.01 or *p* < 0.001; ж, жж or жжж: significantly different from the corresponding db-Rosi respectively with *p* < 0.05, *p* < 0.01 or *p* < 0.001.

6 week-old db/db mice were treated with 8 mg/kg ENOblock (Fig. 2A,B) or 8 mg/kg rosiglitazone over a 24 hour period. Blood glucose level was significantly lowered in ENOblock and rosiglitazone treated mice (Fig. 2C). Consequently, a seven week study of ENOblock treatment in db/db mice was performed (Fig. 2B). Seven weeks treatment with 8 mg/kg or 12 mg/kg ENOblock, or 8 mg/kg rosiglitazone, significantly reduced blood glucose level (Fig. 2D). Rosiglitazone reduced blood glucose level more effectively than ENOblock. After seven weeks, it was observed that ENOblock treatment significantly reduced blood serum LDL cholesterol level, while HDL cholesterol level was unaffected compared to untreated db/db mice (Fig. 2E,F). 12 mg/kg ENOblock treatment in db/db mice also reduced the serum level of free fatty acid (FFA) compared to untreated db/db mice. (Fig. 2G). Serum levels of alanine aminotransferase (ALT) was not significantly changed in ENOblock treated mice compared to untreated db/db mice, indicating that ENOblock treatment did not produce liver hepatocyte toxicity (Fig. 2H).

In the kidney and liver of ENOblock treated mice, enolase activity was significantly reduced. (Fig. 2I,J). In the liver of T2DM mice treated with ENOblock, the known enolase/MBP-1 binding genes, Cox-2^17^, Erbb2 and c-Myc all showed transcriptional repression (Fig. 2K–M).

### ENOblock reduces liver fibrosis and apoptosis in T2DM mice

**Figure 3:**
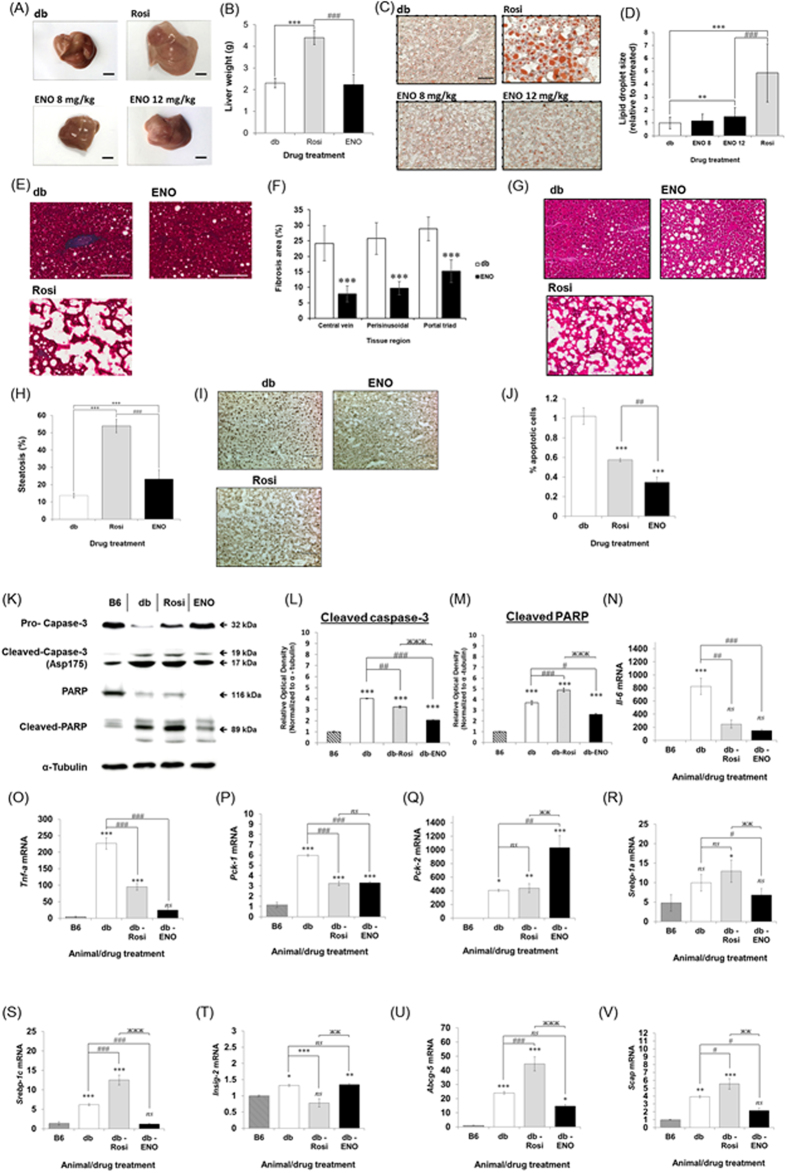
(**A**) Representative photographs of dissected db/db mouse livers 7 weeks after treatment with ENOblock or rosiglitazone. (**B**) Wet liver weight from db/db mice 7 weeks after treatment with 12 mg/kg ENOblock or 8 mg/kg rosiglitazone. (**C**) Oil red O staining for lipid accumulation in liver tissue. (**D**) Quantification of lipid accumulation in treated db/db mice. (**E**) Masson-Trichrome staining for liver fibrosis (blue staining) in the treated mice. Scale bar = 100 μm. (**F**) Quantification of fibrosis after treatment with 12 mg/kg ENOblock. (**G**) H&E staining to detected liver microsteatosis in treated db/db mice. (**H**) Quantification of microsteatosis. (**I**) Labelling of apoptotic cells (black nuclei) in liver tissue from treated mice. Scale bar = 10 μm. (**J**) Quantification of apoptotic cells. (**K**) Western blotting of caspase-3, cleaved caspase-3 (Asp175), PARP and cleaved PARP in liver tissues from treated mice. Age-matched background strain BL6 mice were used for comparison. (**L**) Quantification of cleaved caspase-3 (Asp175) expression relative to α-tubulin. (**M**) Quantification of cleaved PARP expression relative to α-tubulin. (**N**,**O**) Real-time PCR analysis of inflammatory genes Tnf-a and Il-6 in liver tissue. (**P**) Expression of Pck-1 (cytoplasmic form). (**Q**) Expression of Pck-2 (mitochondrial form). (**R**,**S**) Expression of Srebp-1a and -1c. (**T**) Expression of Insig-2 in liver tissue. (**U**,**V**) Expression of LXR target genes Scap and Abcg-5. Statistical analysis for Fig. 3: Values are presented as means ± SD for (**B**,**D**,**F**,**H** and **J**) and ± SE for (**L**–**V**). All statistical analysis was carried out with a one-way ANOVA test, followed by a Dunnett’s multiple comparisons test. *ns*: not significantly different. *, ** or ***: significantly different from the corresponding control (B6) respectively with *p* < 0.05, *p* < 0.01 or *p* < 0.001; #, ## or ###: significantly different from the corresponding db-control respectively with *p* < 0.05, *p* < 0.01 or *p* < 0.001.

Livers were harvested from db/db mice after treatment with ENOblock or rosiglitazone. It was observed that ENOblock treatment did not affect liver weight or gross appearance, whereas rosiglitazone treatment significantly increased liver weight (Fig. 3A,B). Oil red O staining indicated that treatment with 12 mg/kg, but not 8 mg/kg, ENOblock induced lipid accumulation in the liver, although the accumulation was significantly less than 8 mg/kg rosiglitazone treatment (Fig. 3C,D). Masson-Trichrome staining showed that ENOblock treatment significantly reduced liver fibrosis (Fig. 3E,F). Fibrosis in the liver of rosiglitazone treated mice could not be assessed due to the large amount of lipid accumulation (Fig. 2E). To confirm the effect of ENOblock on liver fibrosis, expression of the fibrosis marker, α-smooth muscle actin (α-SMA)^18^, was also assessed. α-SMA expression showed a small but statistically significant decrease in the ENOblock-treated mice compared to untreated db/db mice (Supplementary Figure 2). ENOblock treatment produced an increase in steatosis, although this was significantly less than rosiglitazone treatment (Fig. 3G,H). ENOblock treatment produced a significant reduction in hepatocyte apoptosis and was more effective at inhibiting apoptosis compared to rosiglitazone (Fig. 3I,J). The inhibitory effect of ENOblock treatment on liver cell apoptosis was confirmed by western blotting of two apoptosis markers, cleaved caspase-3^19^ and cleaved PARP^20^ (Fig. 3K–M).

### ENOblock inhibits the expression of inflammatory markers and key regulators of lipid homeostasis and gluconeogenesis in the T2DM liver

ENOblock treatment for seven weeks inhibited the expression of inflammatory markers IL-6 and TNF-α, which are elevated in db/db mice compared to the background B6 strain (Fig. 3N,O). ENOblock was more effective than rosiglitazone at reducing TNF-α expression. Phosphoenolpyruvate carboxykinase-1 (Pck-1cytoplasmic form) has been linked to the positive regulation of gluconeogenesis^21^. Pck-1 expression was down-regulated by ENOblock treatment whereas PEPCK-2 (Pck-2; mitochondrial form), which is not linked to gluconeogenesis regulation, showed increased expression (Fig. 3P,Q). Sterol regulatory element-binding proteins (Srebp-1a and -1C) are major regulators of lipid homeostasis^22^. ENOblock treatment normalized Srebp-1a expression to the level observed in normal B6 mice and also reduced the expression of Srebp-1c (Fig. 3R,S). Insig-2, which regulates Srebp-1 protein activity^23^, was not decreased by ENOblock treatment (Fig. 3T). Liver X receptor (LXR) is a sterol regulated transcription factor that produces hepatic steatosis and hypertriglyceridemia when Srebp-1 is inhibited^24^. However, expression of the LXR target genes Scap and Abcg5 were reduced in the db/db mouse liver after ENOblock treatment (Fig. 3U,V).

### ENOblock reduces adipocyte size and inhibits fibrosis/inflammation in T2DM adipose tissue

**Figure 4:**
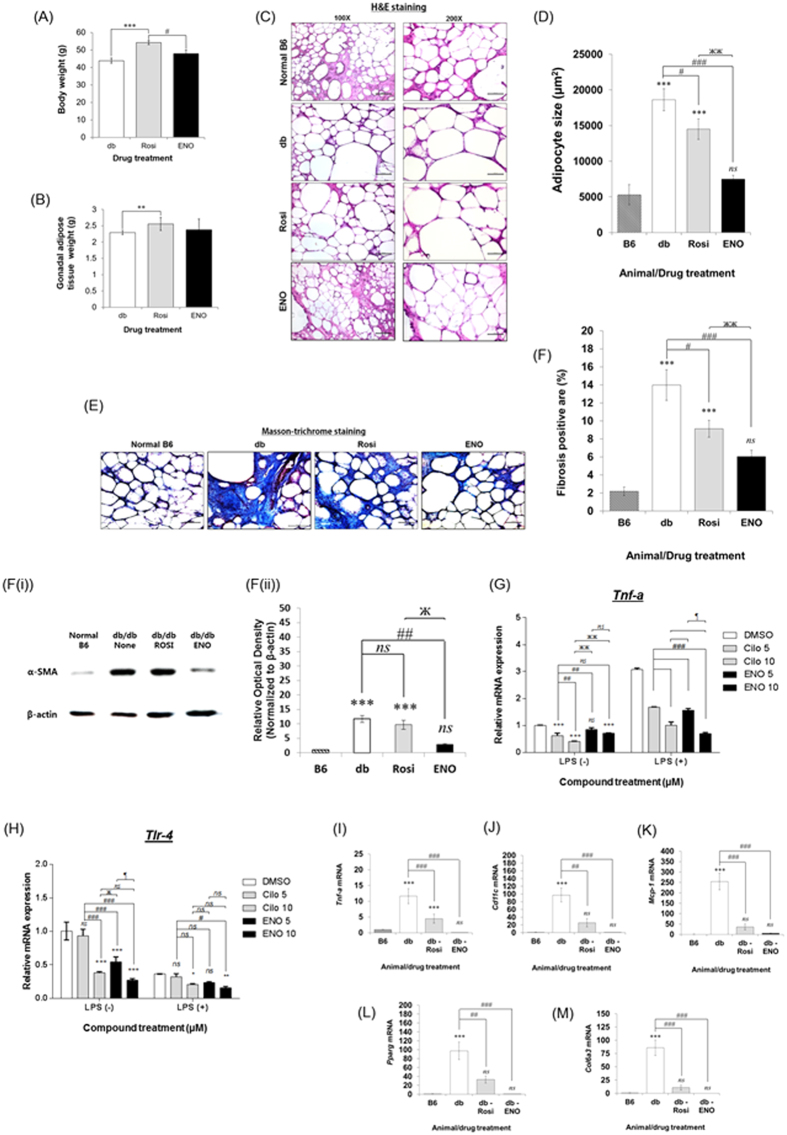
(**A**) Body weight and (**B**) Gonadal adipose tissue weight in db/db mice after 7 weeks treatment with 12 mg/kg ENOblock or 8 mg/kg rosiglitazone. (**C**) H&E staining of gonadal adipose tissue to visualize adipocyte size. Scale bar = 200 μm. (**D**) Quantification of adipocyte size. *p < 0.05 for reduced size compared to untreated. **p < 0.05 for reduced size compared to untreated and rosiglitazone. (**E**) Masson’s trichrome staining of gonadal adipose tissue. Fibrosis areas are visualized as relatively dark blue rings around the adipocyte perimeter, as described^62^. (**F**) Quantification of fibrosis. (**F**(i)) Western blot analysis of the fibrosis marker, α-SMA^18^. (**F**(ii)). Densitometry analysis of α-SMA expression. (**G**,**H**) Effect of ENOblock on LPS-stimulated expression of TNF-α and TLR-4 in RAW 264.7 macrophages. Cells were treated with 5 or 10 μM ENOblock for 16 h before treatment with 100 ng/mL LPS for 3 h. As a positive control, macrophages were pre-treated with 10 or 20 μM cilostazol, an anti-platelet drug that ameliorates insulin resistance by suppressing chronic inflammation^25^. (**I**–**M**) Expression of the inflammatory genes Tnf-a, Cd11c and Mcp-1, the adipogenesis marker Pparg, and the marker of adipose fibrosis col6a3, in adipose tissue. Statistical analysis: values are presented as means ± SD for (**A**,**B**,**D**,**F** and **G**,**H**) and ± SE for (**I**–**M**). (**G** and **H**) Statistical analysis was carried out with a two-way ANOVA test, followed by a Sidak’s mutiple comparisons test. *ns*: not significantly different. *, ** or ***: significantly different from the corresponding DMSO-control respectively with p < 0.05, p 0.01, p < 0.001; #, ## or ###: significantly different from the corresponding Cilo 5 respectively with p < 0.05, p < 0.01 or p < 0.001; ж, жж or жжж: significantly different from the corresponding Cilo 10 respectively with p < 0.05, p < 0.01 or p < 0.001; ¶, ¶¶ or ¶¶¶: significantly different from ENO 5 respectively with p < 0.05, p < 0.01 or p < 0.001. (**A**–**F** and **I**–**M**) Statistical analysis was carried out with a one-way ANOVA test and Dunnett’s multiple comparisons. *ns*: not significantly different. *, ** or ***: significantly different from the B6 control with *p* < 0.05, *p* < 0.01 or *p* < 0.001; #, ## or ###: significantly different from the db-control respectively with *p* < 0.05, *p* < 0.01 or *p* < 0.001. Error = SEM.

ENOblock treatment did not produce an increase in total body weight, compared to rosiglitazone treatment, although there was no difference in gonadal adipose tissue weight compared to rosiglitazone treated mice (Fig. 4A,B). The db/db mice showed increased adipocyte size and adipose fibrosis compared to the non-diabetic, C57BL/6J (B6) background strain (Fig. 4C–F). Compared to untreated db/db mice, ENOblock significantly decreased adipocyte size and adipose tissue fibrosis (Fig. 4C–F). ENOblock treatment also decreased expression of the fibrosis marker, α-smooth muscle actin^18^ (Fig. 4F(i,ii)). The measurement of inflammatory gene induction in macrophages has been used to evaluate compounds that suppress inflammation in adipose tissue^25^. ENOblock inhibited the induction of inflammatory genes TNF-α and toll-like receptor 4 is (TLR4) in macrophages stimulated with pro-inflammatory LPS (Fig. 4G,H). The inhibitory effect of ENOblock on TLR4 expression was greater than cilostazol, an inhibitor of phosphodiesterase 3B that ameliorates insulin resistance by suppressing chronic inflammation in T2DM adipose tissue^25^. In gonadal adipose tissue, real time PCR showed that ENOblock treatment down-regulated expression of the inflammatory genes TNF-α, monocyte chemoattractant protein 1 (MCP-1) and pro-inflammatory macrophage marker CD11c (Fig. 4I–K). The nuclear receptor peroxisome proliferator-activated receptor-γ (Pparg), which is a marker of increased adipogenesis in T2DM adipose tissue^25^, showed decreased expression after ENOblock treatment (Fig. 4L). Collagen VI, which has been linked to metabolic dysregulation and adipose tissue fibrosis^26^, was also down-regulated by ENOblock treatment (Fig. 4M).

### ENOblock reduces cardiac hypertrophy, fibrosis, apoptosis and pathological markers in T2DM mice

**Figure 5:**
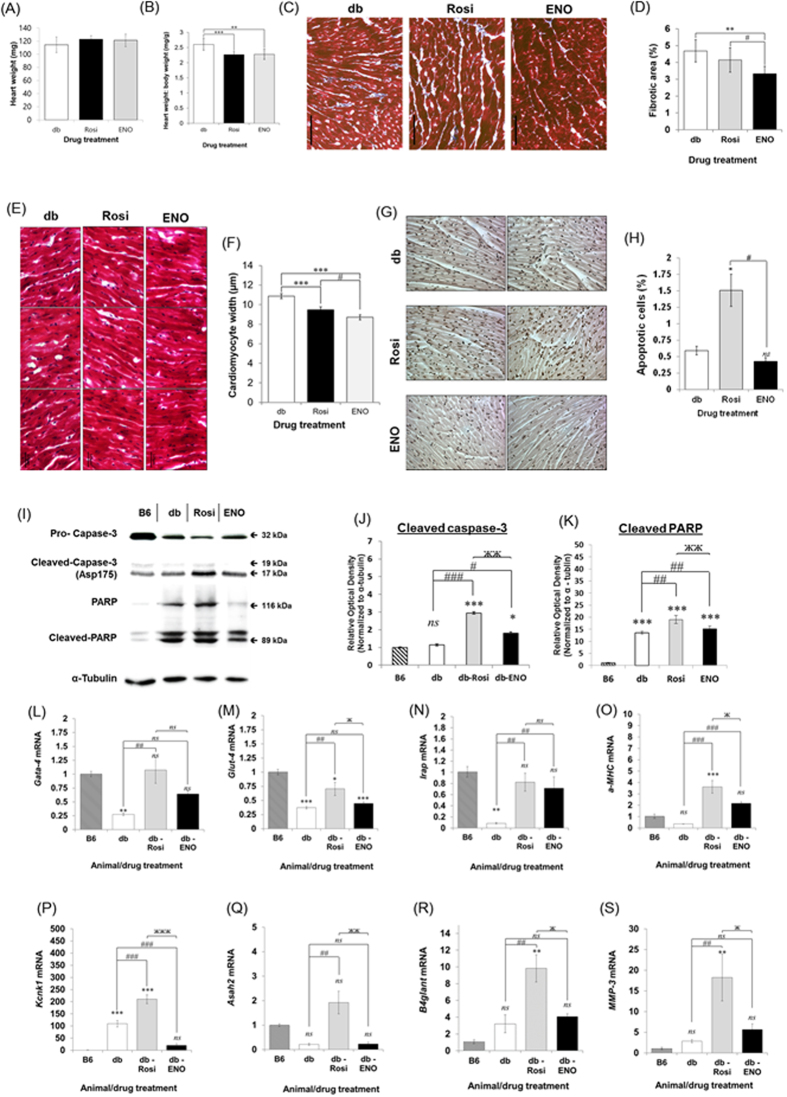
(**A**) Heart weight in db/db mice treated with 8 mg/kg rosiglitazone or 12 kg/mg ENOblock for 7 weeks. (**B**) Ratio of body weight to heart weight (an indicator of cardiac hypertrophy). (**C**) Masson-trichrome staining of cardiac tissue to detect fibrosis (blue staining). Scale bar = 100 μm. (**D**) Quantification of cardiac fibrosis. (**E**) Representative images of cardiomyocytes visualized by H&E staining. (**F**) Measurement of cardiomyocyte width. (**G**) Staining of apoptotic nuclei in cardiac tissue from treated mice. Scale bar = 100 μm. (**H**) Quantification of apoptotic cells in cardiac tissues. (**I**) Western blotting of caspase-3, cleaved caspase-3 (Asp175), PARP and cleaved PARP in heart tissue from the treated mice. Age-matched background strain BL6 mice were used for comparison. (**J**) Quantification of cleaved caspase-3 (Asp175) expression relative to α-tubulin. (**K**) Quantification of cleaved PARP expression relative to α-tubulin. (**L**–**O**) Real-time PCR analysis of Gata-4, Glut-4, Irap and a-MHC, which are markers of cardiac function^27^. (**P**–**S**) Real-time PCR analysis of Kcnk-1, Asah-2, B4glant and MMP-3, which are markers of cardiac pathology^29^. Statistical analysis: values are presented as means ± SD for (**A**,**B**,**D** and **F**); ±SE for (**H**) and (**J**–**S**). All statistical analysis was carried out with a one-way ANOVA test, followed by a Dunnett’s multiple comparisons test. *ns*: not significantly different. *, ** or ***: significantly different from the corresponding control (B6) respectively with *p* < 0.05, *p* < 0.01 or *p* < 0.001; #, ## or ###: significantly different from the corresponding db-control respectively with *p* < 0.05, *p* < 0.01 or *p* < 0.001. Error = SEM.

Heart weight was unaffected by ENOblock treatment (Fig. 5A). The body weight: heart weight ratio, an indicator of cardiac hypertrophy^27^, was decreased by ENOblock treatment (Fig. 5B). Masson-Trichrome staining indicated that ENOblock treatment reduced cardiac fibrosis (Fig. 5C,D) and cardiomyocyte hypertrophy (Fig. 5E,F). ENOblock also reduced apoptosis in cardiac tissue, in contrast to rosiglitazone, which increased apoptosis (Fig. 5G,H). The lower level of apoptosis in ENOblock treated cardiac tissue, compared to rosiglitazone, was confirmed by western blotting of the apoptosis marker, cleaved PARP^20^ and cleaved caspase-3^19^ (Fig. 5I–K). Gata-4, glucose transporter-4 (Glut-4), insulin-regulated aminopeptidase (Irap) and α-myosin heavy chain (α-MHC) are markers of cardiac function^28^. ENOblock treatment increased the expression of Gata-4, Irap and α-MHC, but not Glut-4 (Fig. 5L–O). Potassium two pore domain channel subfamily K member 1 (Kcnk1), N-acylsphingosine amidohydrolase 2 (Asah2), beta-1,4-N-acetyl-galactosaminyl transferase 1 (B4glant) and matrix metalloproteinase 3 (MMP-3) are markers of cardiac pathology^29^. Rosiglitazone treatment increased the expression of these markers compared to untreated db/db mice. ENOblock treatment reduced the expression of Kcnk1 compared to untreated db/db mice and reduced the expression of Asah2, B4glant and MMP-3 compared to the mice treated with rosiglitazone (Fig. 5P–S).

### ENOblock reduced fibrosis and apoptosis in T2DM kidney tissue and reduced the expression of Pck-1

**Figure 6:**
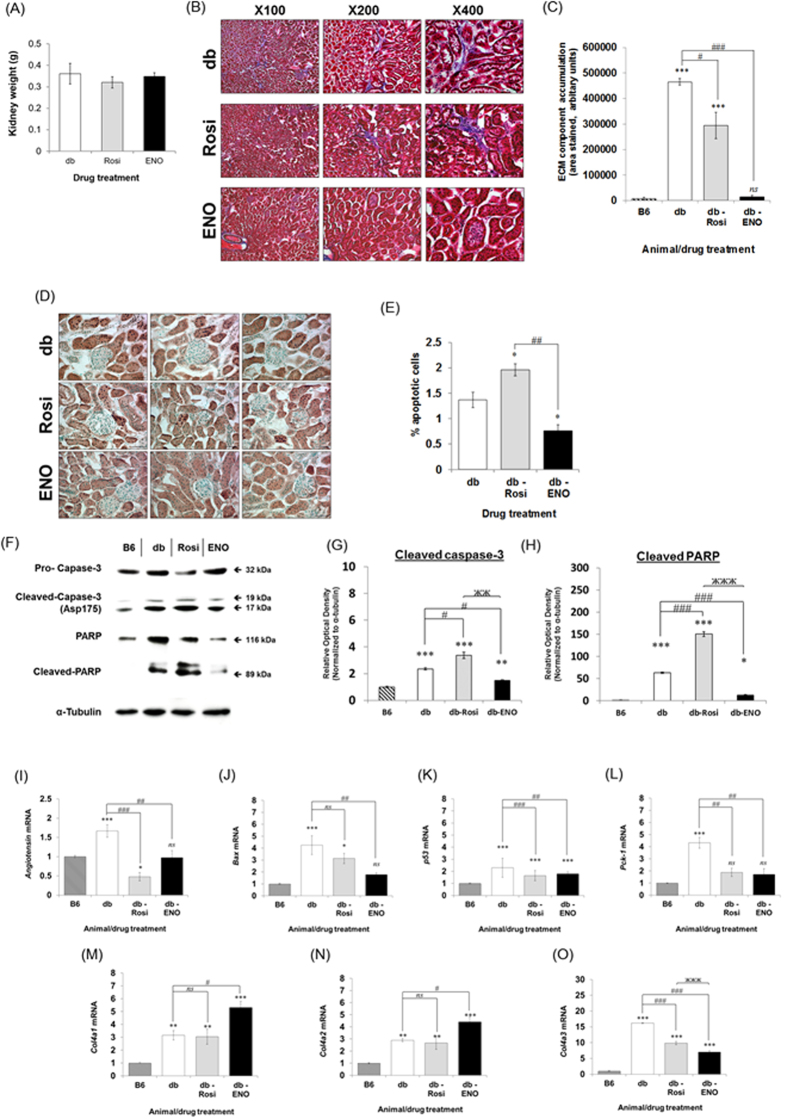
(**A**) Kidney weight after 7 weeks treatment with 12 mg/kg ENOblock or 8 mg/kg rosiglitazone. (**B**) Masson’s trichrome staining of kidney tissue to detect fibrosis (blue staining). (**C**) Quantification of interstitial ECM component accumulation. (**D**) Staining of apoptotic nuclei in kidney tissue. Scale bar = 100 μm. (**E**) Quantification of apoptotic cells in kidney tissues. (**F**) Western blotting of caspase-3, cleaved caspase-3 (Asp175), PARP and cleaved PARP in the kidney tissue from treated mice. Age-matched background strain BL6 mice were used for comparison. (**G**) Quantification of cleaved caspase-3 (Asp175) expression relative to α-tubulin. (**H**) Quantification of cleaved PARP expression relative to α- tubulin. (**I**–**L**) Real-time PCR analysis of angiotensin, Bax and p53, which are makers of kidney cell apoptosis, and Pck-1 (cytoplasmic), which is linked to the positive regulation of gluconeogenesis^21^,^30^. (**M**–**O**) Expression of Col4a1, Col4a2 and Col4a3 in kidney tissue. Statistical analysis: values are presented as means ± SD for (**A**) and ± SE for (**C**,**E** and **G**–**L**). All statistical analysis was carried out with a one-way ANOVA test, followed by a Dunnett’s multiple comparisons test. *ns*: not significantly different. *, ** or ***: significantly different from the corresponding control (B6) respectively with *p* < 0.05, *p* < 0.01 or *p* < 0.001; #, ## or ###: significantly different from the corresponding db-control respectively with *p* < 0.05, *p* < 0.01 or *p* < 0.001. Error = SEM.

ENOblock treatment did not significantly affect kidney weight (Fig. 6A). Masson-trichrome staining indicated that ENOblock reduced kidney fibrosis (Fig. 6B,C). In addition. ENOblock treatment reduced apoptosis, whereas rosiglitazone treatment increased apoptosis (Fig. 6D,E). This inhibitory effect of ENOblock treatment on kidney cell apoptosis was confirmed by western blotting of two apoptosis markers, cleaved caspase-3^19^ and cleaved PARP^20^ (Fig. 6F-H). The genes angiotensin, Bax and p53 have been used as markers of apoptosis in the T2DM kidney^30^. Real-time PCR analysis indicated that ENOblock treatment reduced expression of these three genes compared to untreated mice (Fig. 6I–K). The kidney is a major site of gluconeogenesis^31^. ENOblock down-regulated the expression of Pck-1 (cytoplasmic form), which has been linked to the positive regulation of gluconeogenesis (Fig. 6L)^21^. Collagen 4a3 is glomerulus-specific and excess levels have been linked to glomerular nephritis, whereas collagen 4a1 and collagen 4a2 are ubiquitously expressed in basement membranes^32^. ENOblock treatment reduced the expression of collagen IVa3 and increased the expression of collagens IVa1 and IVa2 (Fig. 6M–O).

## Discussion

In this study, we have investigated the novel enolase targeting molecule, ENOblock, as a potential new strategy for treating T2DM. Enolase possesses numerous ‘moonlighting’ roles in cells that are not related to its glycolytic activity. ENOblock was previously used to show that enolase regulated glucose uptake and expression of Pck-1 in cells and zebrafish^9^. Herein, we show for the first time that ENOblock reduces enolase activity and produces numerous beneficial effects in a mammalian model of T2DM.

Our mechanistic studies for ENOblock indicate that this compound decreases enolase activity and increases the nuclear localization of enolase, where it can function as a transcription repression (Fig. 1A–E)^15^. This was not observed using the enzymatic inhibitor, NaF, (Fig. 1L–M), suggesting that ENOblock modulates the non-glycolytic ‘moonlighting’ functions of enolase to increase nuclear localization. This phenomena has been observed for the small molecule, GAPDS, which increases the nuclear localization of the glycolysis enzyme, GAPDH, to regulate gene expression^33^. Suppression of the known enolase-binding gene, c-Myc, was not observed after ENOblock treatment in Huh7 hepatocytes, although this may be due to the cell line used in this study (Huh 7), which is a cancer line with dysregulated c-Myc regulation^34^. In support of this, expression of the known enolase-binding genes, c-Myc, Erbb2 and Cox-2, were all down regulated in the liver of ENOblock treated mice (Fig. 2K-M).

Our study used the known anti-diabetes drug, rosiglitazone as a positive control for comparison with enolase. We selected rosiglitazone for comparison with ENOblock in T2DM mice due to three considerations: (i) it was previously studied alongside ENOblock in cell based assays, (ii) Rosiglitazone and ENOblock were shown to inhibit Pck-1 expression, which can regulate gluconeogenesis^9^,^21^, (iii) Rosiglitazone produces significant side effects^5^,^35^, which can be compared with ENOblock treatment. Over seven weeks treatment, rosiglitazone was more effective than ENOblock at reducing blood glucose level (Fig. 2D). In contrast, 12 mg/kg ENOblock was more effective than 8 mg/kg rosiglitazone at reducing blood LDL cholesterol level, while maintaining HDL cholesterol level, which increases cardioprotection (Fig. 2E,F)^36^. When comparing 12 mg/kg ENOblock and 8 mg/kg rosiglitazone, it should be noted that the micromolar dose for rosiglitazone is higher than ENOblock: 22.4 mM and 20.2 mM, respectively. In the db/db mouse liver, ENOblock produced less lipid accumulation and steatosis than rosiglitazone (Fig. 3A–H). Although hepatosteatosis after is only rarely reported in humans treated with rosiglitazone, it has been observed in rodent models of diabetes^37^. 12 mg/kg ENOblock treatment produced greater reductions in blood LDL/VLDL cholesterol compared to rosiglitazone treated mice (Fig. 2(E,F)). In addition, the 12 mg/kg ENOblock treatment decreased the serum level of free fatty acid compared to untreated db/db mice (Fig. 2G). This could be due to the observed inhibition of Srebp-1a and -1c, which are key regulators of lipid homeostasis (Fig. 3R,S)^23^. Interestingly, betulin, a small molecule present in birch (*Betula*) tree bark, improved hyperlipidemia and insulin resistance by targeting Srebp-1^24^. Whereas betulin inhibits Srebp-1 signaling via blocking protein cleavage and activation, ENOblock reduces Srebp-1 expression. This finding is supported by lack of effect of ENOblock on Insig-2 expression, which binds Srebp-1 protein and inhibits its activation^24^. However, betulin is poorly soluble and not suitable for drug development^38^. This inhibitory effect of ENOblock on Srebp-1 may also explain the reduced adipocyte size observed in treated db/db mice (Fig. 4C,D). ENOblock inhibited Srebp-1 without up-regulating LXR target genes (Fig. 3U,V). This specific inhibition of Srebp-1 without activating LXR should inhibit both cholesterol and fatty acid biosynthesis and may be applicable for treating both atherosclerosis and T2DM^24^. It should also be noted that ENOblock treatment does not appear to cause liver toxicity, as indicated by unchanged levels of serum ALT compared to untreated db/db mice (Fig. 2H).

ENOblock treatment also produced anti-fibrotic effects in the liver, adipose tissue, heart and kidney (Figs 3E,F, 4E,F, 5C,D and 6B,C). Tissue inflammation is linked to fibrosis^39^ and ENOblock treatment reduced the expression of inflammatory markers in liver (Fig. 3N,O), adipose tissue (Fig. 4I–K) and activated macrophages (Fig. 4G,H), which may explain these anti-fibrotic effects. Enolase is known to ‘moonlight’ as a regulator of the inflammatory response by functioning as a plasminogen receptor on the cell surface^15^. Thus, the effect of ENOblock treatment on the interaction between enolase and plasminogen could be a rewarding area for further investigation.

In the heart, ENOblock did not increase apoptosis to the degree observed after rosiglitazone treatment (Fig. 5G–K). The use of rosiglitazone to treat human T2DM was restricted due to increased risk of myocardial infarction^5^. However, there have been conflicting reports on the effect of rosiglitazone on cardiac apoptosis^40^,^41^,^42^. To our knowledge, our data is the first visualization of rosiglitazone-induced apoptosis in the heart. Compared to rosiglitazone, ENOblock treatment showed reduced expression of genes linked to cardiac pathology (Fig. 5P–S). Therefore, based on this data, ENOblock may not produce the cardiac issues linked to rosiglitazone treatment. The study by Broderick *et al*. showed that Gata-4 expression is reduced in the hearts of db/db mice and this is thought to contribute to hyperglycemia-induced cardiomyocyte injury^28^,^43^. The same study also demonstrated down-regulation of the structural and contractile genes α-MHC, Irap and the co-secreted Glut-4^28^. Our results show that Gata-4, α-MHC and Irap expression in the db/db mouse heart was increased after ENOblock treatment (Fig. 5L,N,O). The study by Wilson *et al*. showed that four genes linked to cardiac pathology are up-regulated in the cardiac tissue of db/db mice treated with rosiglitazone: Kcnk1, Asah2, B4glant and MMP-3^29^. ENOblock significantly reduced the expression of Kcnk1, Asah2, and did not increase B4glant and MMP-3 expression compared to the db/db mice (Fig. 5P–S). The up-regulation of Gata-4, α-MHC and Irap, without increasing Kcnk1, Asah2, B4glant and MMP-3 expression, may explain the beneficial effects of ENOblock on cardiomyocyte hypertrophy and fibrosis in the db/db heart.

A previous study by Verschuren *et al*. reported that mice fed a high fat diet to induce T2DM and treated with rosiglitazone showed a significant increase in heart weight^44^. We speculate that the differences in heart weight gain observed in our study and Verschuren *et al*. may be due to differences in the animal model of T2DM. The study by Wilson *et al*., which we referenced for assessing makers of cardiac disease pathology, reported that rosiglitazone treatment in db/db mice produced an increase in body weight and a downward trend in the heart:body weight ratio^29^. Morphological analysis of whole hearts also indicated that there was no weight gain after rosiglitazone treatment^29^. Therefore, we speculate that rosiglitazone treatment can induce heart weight gain in the high fat diet model, but not the db/db model.

ENOblock treatment produced relatively strong effects in the T2DM kidney, with both fibrosis and apoptosis reduced in comparison with untreated mice, along with reduced expression of glomerulus-specific collagen type 4a3 (Fig. 6O). In contrast, rosiglitazone increased apoptosis. To our knowledge, this is the first report of increased apoptosis linked to rosiglitazone treatment in T2DM mice. The beneficial effects of ENOblock in the kidney of T2DM mice may warrant further investigation of this compound in other models of kidney dysfunction.

We speculate that the drop in enolase activity is not responsible for the gene expression changes observed after ENOblock treatment, although this cannot be ruled-out in this study. Reduced enolase activity should inhibit glycolysis, which would produce a decrease in cellular glucose uptake. However, ENOblock treatment produces an increase in glucose uptake and anti-diabetic effects, which we speculate is cause by down-regulation of the gluconeogenesis-regulatory gene, Pck-1^21^, or down-regulation of Srebp-1^24^,^45^. Enolase is known to act as a transcriptional repressor in the nucleus. However, enolase target genes in the nucleus are being characterized. For example, the recent study by De Rosa *et al*. showed that enolase represses Foxp3 expression^46^. Techniques such as ChIP-seq are required to fully characterize the target genes for enolase. We believe that this type of analysis would be an interesting area for future study.

α-Enolase (ENO1) can be alternatively translated into a 37 kD isoform known as Myc-binding protein-1 (MBP-1), which lacks the glycolytic activity of enolase. MBP-1 occurs in the nucleus and functions as a tumor suppressor by inhibiting the *c-myc* proto-oncogene promoter^47^. To our knowledge, the potential role of MBP-1 in diabetes has not been investigated. The antibody used in our analysis of enolase expression does not detect a band at 37 kD, suggesting that it does not detect MBP-1. This indicates that the effect of ENOblock on MBP-1 is currently unknown and should be an important area for further investigation.

Different classes of drugs have been developed to treat T2DM. Two newer examples are glucagon-like peptide-1 receptor (GLP-1) agonists (such as exenatide, liraglutide, and lixisenatide) and the gliflozin/SGLT2 inhibitor class of drugs (including dapagliflozin, empagliflozin and canagliflozin)^48^,^49^,^50^. Both classes of drug have been shown to improve metabolic parameters in humans, such as body weight, serum lipid profile and cardiovascular outcome^49^,^50^,^51^. In T2DM mice, treatment with GLP-1 agonists has been shown to reduce diabetic nephropathy, improve cardiac function and reduce liver damage^52^,^53^,^54^. Similar effects were observed after treatment with SGLT2 inhibitors^55^,^56^,^57^. In our study, ENOblock treatment improved markers of cardiac, liver and kidney dysfunction and was compared with rosiglitazone in the db/db T2DM model. Although both drugs produced anti-diabetic effects, there were differences in their effects in liver tissue (e.g. liver weight and Srebp-1 expression), heart (e.g. apoptosis level and expression of damage markers Kcnk1/Asah2/B4glant/MMP-3) and kidney (e.g. apoptosis and fibrosis levels). As a further study, it would be interesting to compare the action of ENOblock with GLP-1 agonists or SGLT1 inhibitors in the susceptible tissues of T2DM animal models.

Previously, ENOblock has been shown to bind enolase and inhibit its activity^9^. However, enolase has numerous additional ‘moonlighting’ functions in cells that are not related to its enzyme activity^15^. One of these functions is gene regulation after increased localization to the nucleus^46^,^58^. In this study, we have also shown that ENOblock treatment increases the nuclear localization of enolase. Therefore, our proposed mechanism for ENOblock is the modulation of enolase target gene expression. Further characterization of the enolase target genes that produce the anti-diabetic effect of ENOblock, using techniques such as ChIP seq, should be an interesting area for further study.

In summary, the enolase binding compound, ENOblock, was found to produce beneficial effects in murine T2DM and this study is the first reported test of this compound in a mammalian disease model. The effects of ENOblock treatment in murine T2DM are shown in Table 1. ENOblock alleviated hyperglycemia, hyperlipidemia, tissue apoptosis and fibrosis. Down-regulated expression of Pck-1 and Srebp-1 by ENOblock treatment can explain the anti-diabetic effects of ENOblock in T2DM mice. ENOblock treatment increased nuclear localization of enolase and decreased its activity *in vivo*. ENOblock may be used as a chemical probe to characterize the effects of enolase inhibition in different disease models. Given the small number of reported enolase-binding small molecules, this study supports the development of ENOblock as a novel drug candidate for T2DM. Once considered as a ‘boring’ housekeeping enzyme^15^, this study also implicates enolase as a novel drug target for T2DM.

## Methods

### Reagents and antibodies

ENOblock (N-[2-[2-(2-aminoethoxy)ethoxy]ethyl]-4-[[4-[(cyclohexylmethyl)amino]-6-[[(4-fluorophenyl)methyl]amino]-1,3,5-triazin-2-yl]amino]-benzeneacetamide hydrochloride) was a kind gift from Professor Young-Tae Chang, National University of Singapore. Rosiglitazone was purchased from Santa Cruz Biotechnology (CA, USA). NaF, oil red O and lipopolysaccharide (LPS) and an antibody for α-smooth muscle actin (catalogue number A5228) was purchased from Sigma-Aldrich (MO, USA). Cilostazol was purchased from Enzo Life Sciences (NY, USA). Antibodies for enolase (catalogue number sc271384), lamin B (catalogue number sc374015), pro-caspase-3 (catalogue number sc7148) and α-tubulin (catalogue number sc53646) were purchased from Santa Cruz Biotechnology. An antibody for histone H3, cleaved caspase-3 (catalogue number cs-#9661) and PARP/cleaved PARP (catalogue number cs-#9542) was purchased from Cell Signaling (catalogue number #9715). Nonident-P40 (IGEPAL CA-630) was purchased from Generay Biotech.

### Cell culture

NIH/3T3 murine fibroblasts, Huh7 human hepatocytes, 3T3-L1 murine pre-adipocytes and RAW 264.7 murine macrophages were obtained from the Korean Cell Line Bank (KCLB) - Seoul National University, Republic of Korea. Cells were maintained in proliferation media, consisting of DMEM supplemented with 10% FBS, 50 units mL^−1^ penicillin and 50 mg mL^−1^ streptomycin.

### Animal study

The study conformed to the Institute for Laboratory Animal Research Guide for the Care and Use of Laboratory Animals and was approved by the Gwangju Institute of Science and Technology Animal Care and Use Committee (approval number: GIST-2016-33). Male C57BL/Ksj-db/db mice were purchased from Damool Science (Dae-Jeon, Republic of Korea) at 5 weeks of age. The mice were maintained in a 12 h/12 h light/dark cycle. Food and water were freely available. After an acclimation period of 1 week, the mice were used for experiments. For comparison with the normal background strain, male C57BL/6J mice were also purchased from Damool Science and age-matched for comparison with the drug treated C57BL/Ksj-db/db mice.

For the short-term test to evaluate the anti-hyperglycemic effect of ENOblock, mice were randomly divided into 3 groups of 10 mice. Vehicle (10% DMSO in saline), rosiglitazone (8 mg/kg in saline with 10% DMSO) and ENOblock (8 mg/kg in saline with 10% DMSO) were administrated by intraperitoneal injection (solution volume = 10 uL/g) and blood glucose level was measured immediately for the 0 h time-point. Blood glucose levels were then measured after 1, 3, 6, 12 and 24 h.

For the 7 week study of ENOblock treatment, db/db mice were randomly divided into 4 groups of 10 mice. Vehicle (10% DMSO in saline), rosiglitazone (8 mg/kg in saline with 10% DMSO) or ENOblock (8 mg/kg or 12 mg/kg in saline with 10% DMSO) were administrated by intraperitoneal injection (solution volume = 10 μL/g.) every 24 h. Blood glucose monitoring was carried out every 48 h during the 7 weeks of drug treatment (on the day of blood collection, it was carried out 6 h after the drug treatment). Blood glucose was measured using the OneTouch Ultra (LifeScan, CA, USA). After 7 weeks, mice were sacrificed by the inhalation of diethyl ether. Blood was collected from the heart, and the kidneys, liver gonadal adipose tissue were harvested. The collected blood was placed in a 1.5 mL tube for clotting at room temperature for 15 min. The clot was removed by centrifugation at 1500 *g* for 10 min at 4 °C. The supernatant was separated into 50 μL aliquots and stored at −80 °C. Harvested tissues were washed two times in PBS and stored at −80 °C. Blinding was carried out for the following tissue analyses: real-time PCR, fibrosis staining, apoptosis assay and enolase activity assay.

### Tissue embedding and sectioning

Tissues were washed two times with PBS, blotted dry, placed in a cryo-mold and covered completely with OCT (Leica, Germany). OCT-embedded samples were snap-frozen by floating on liquid nitrogen and placed in an isopropanol slurry. Frozen blocks were further stored at −80 °C until sectioned. Every section was made with a Leica CM 1850 cryostat at −25 °C.

### Assessment of cardiac myocyte width

Frozen heart tissue from five mice in each treatment group was sectioned at an 8 μm depth, and fixed using 3.7% formaldehyde solution. Hematoxylin and eosin (H&E) staining were performed to measure cardiac myocyte width. Myocyte width was measured in regions of myocardium with parallel myocyte fascicles by using Aperio ImageScope (Leica Biosystems, Germany). Between 30 and 47 myocytes from each heart was measured.

### Extraction of nuclear proteins

Cells were cultured and treated with compound of interest in 10 cm dishes. After treatment, the cells were washed with PBS and cytosolic extraction buffer was added (10 mM Hepes pH 7.9, 10 mM KCl, 0.1 mM EDTA, 0.5% nonident-P40 (IGEPAL CA-630, which does not disrupt the nuclear membrane) and protease inhibitor cocktail). Cells were collected by scraping and centrifuged at 3000 rpm at 4 °C for 5 minutes, after 5 minutes incubation on ice. The supernatants were discarded and nuclear pellets were washed twice with detergent-free cytosolic extraction buffer. One pellet volume of nuclear extraction buffer (20 mM Hepes pH 7.9, 0.4 M NaCl, 1 mM EDTA, 25% glycerol and protease inhibitor cocktail) was added, followed by incubation for 15 minutes on ice with occasional vortexing. Nuclear proteins were centrifuged at 13000 rpm at 4 °C for 5 minutes and the supernatants were collected.

For the data in Fig. 1O,P and Supplementray Fig. 1, nuclear proteins were prepared from cells and tissues using the Nuclear/Cytosol Fractionation Kit (#K266-25, BioVision Incorporated, CA 95035 USA).

### Primary mouse hepatocyte purification

Primary hepatocytes were isolated from 10 weeks old male C57BL/6J mouse for the enolase translocation assay. The protocol was modified to add the percoll gradient method from a two-step collagenase perfusion procedure to isolate hepatocytes, as described by Seglen *et al*.^59^. The cells were plated in DMEM medium supplemented with 10% FBS, 10 μM dexamethasone, 100 nM insulin and 1% P/S, at a density of 2 × 10^6^ cells/plate in a 100 mm diameter cell culture plate.

### Enolase activity assay

Enolase activity was tested by detecting the transformation of 2-phosphoglycerate to phosphoenolpyruvate (Enolase Activity Colorimetric Assay Kit, Biovision, CA, USA). Homogenized liver tissue (10 mg) from 6 animals per treatment group in triplicate was used for the tissue-based assay. Cells (2 × 10^5^) were treated in triplicate for the cell-based assay. Enolase activity was measured at an absorbance of 570 nm in the kinetic mode after 20 min.

### *In vitro* enolase nuclear translocation assay

Primary mouse hepatocytes in 100 mm diameter cell culture plates (2 × 10^6^ cells/plate) were maintained DMEM medium with 10 μM ENOblock or 0.1% DMSO for 72 h. Nuclear and cytoplasmic proteins were isolated using the Nuclear/Cytosol Fractionation Kit (Biovision Inc.). The protein samples were prepared in triplicate for western blot assay.

### *In vivo* enolase nuclear translocation assay

C57BL/6J mice (8 weeks old) were injected intraperitoneally with either 12 mg/kg ENOblock in saline plus 10% DMSO or saline plus 10% DMSO (5 mice per treatment group). After 24 hours, the mice were sacrificed and liver tissue was harvested. Nuclear and cytoplasmic proteins were isolated from the liver tissue using the Nuclear/Cytosol Fractionation Kit (Biovision Inc.).

### Western blotting

Extracts of 30 to 40 μg protein samples were loaded onto 10% polyacrylamide gels, and transferred to nitrocellulose membranes after electrophoresis. Details of the primary antibodies used are provided in the Reagents and Antibodies section. Detection was performed using a HRP-conjugated secondary (anti-mouse IgG-HRP, sc2031, Santa Cruz Biotech). All primary antibodies were used at a 1:1000 dilution in TBS-T with 5% skimmed milk and incubated with membranes overnight at 4 °C. The secondary antibody was used at a 1: 10000 dilution and incubated for 30 min at room temperature (RT). The concentration of proteins was measured using the Bradford assay. Expression signals were visualized using an ECL solution (RPN2232, GE Healthcare Life Science, UK). Band intensity was measured by ImageJ 1.45 s software (National Institutes of Health, USA) and normalized with α-tubulin for cytosolic proteins and lamin B for the nuclear extraction. Protein samples from the tissues of five mice in each treatment group were used for western blotting analysis.

### RNA extraction from cells and tissues

RNA was harvested using the TRIzol reagent (Thermo Fisher Scientific, MA, USA), following the manufacturer’s instructions. Tissues frozen in liquid nitrogen were ground into powder using a glass mortar prior to RNA extraction.

### RT-PCR and quantitative real-time PCR

RNA was harvested from cells or tissues using the TRI reagent, following the manufacturer’s instructions (Sigma-Aldrich, MO, USA). For PCR analysis in tissues, RNA was harvested from 5 mice in each treatment group. For RT-PCR, 0.5 μg RNA and 100 pmole oligo dT16 was used for reverse transcription (AccuPower^^®^^ RT PreMix; Bioneer). 2.5 μL RT product was used for the PCR (AccuPower^®^ PCR PreMix; Bioneer). Densitometry analysis was performed using the Scion Image program (Scion Corporation, USA). For quantitative real-time PCR, the transcript level of genes of interest was analyzed by using the StepOnePlus Real Time PCR System (Applied Biosystems, UK). Total RNA was reverse transcribed to prepare cDNA using the AccuPower^®^ RT PreMix (Bioneer Corporation). The cDNA obtained was subjected to real-time PCR according to the manufacturer’s instructions with the following modifications: PCR was performed in triplicate in a total volume of 20 μL 2X Power SYBR^®^ Green PCR Master Mix(Applied Biosystems, UK) containing each 200 nM (final concentration) of the specific primer and 1 μL of cDNA. PCR amplification was preceded by incubation of the mixture for 10 min at 95 °C and the amplification step consisted of 40 cycles of denaturation, annealing and extension. The denaturation was performed for 15 s at 95 °C, annealing was done for 1 m at 60 °C and the extension was performed at 72 °C for 20 s with fluorescence detection at 72 °C after each cycle. After the final cycle, melting-point analysis of all of the samples was performed within the range of 60–95 °C with continuous fluorescence detection. A specific cDNA sample was included in each run and served as a reference for the comparison between runs. The expression level of actin or 18s rRNA was used for normalization while calculating the expression levels of all of the other genes (as indicated in the text). Results were expressed as the relative expression level for each gene. The synthesized cDNAs were amplified by quantitative real time PCR (qRT-PCR) or standard PCR. The PCR primers used in this study are shown in Supplementary Table 1. To measure the relative amount of mRNA expression in each experiment, firstly, the Δ CT value of each gene is calculated with respect to the CT values of β-actin or 18S rRNA (internal control) in each cDNA sample. Next, the Δ CT value of each gene in the drug treated samples were further normalized with the Δ CT value of the same gene in the non-treated db/db sample or normal healthy control (B6, same age with experimental group) to calculate the ΔΔ CT value, indicating the relative expression of each gene in the drug treated samples compared with that of the controls. The relative mRNA expression fold-change was finally calculated using the 2^−2ΔΔCT^ method. The final mRNA expression in each experiment is calculated as the average value of three independent set of experiments.

### Serum Free fatty acid (FFA) quantification

Blood samples were centrifuged at 10, 000 *g* for 15 min at 4 °C and the serum (supernatant) collected into new tubes and frozen at −80 °C until the time of FFA analysis. The concentration of FFA was measured using a kit (catalog #K612, BioVision, Inc.), using the colormetric assay (OD 570 nm) and expressed as nmol/μl or mM. Blood serum samples were used from 6 animals per treatment group in triplicate.

### Serum Alanine aminotransferase (ALT) activity assay

Serum was prepared as described for the FFA quantification. Enzyme activity was expressed as nmol/mon/mL(=mU/mL) the and assay was carried out in accordance with the method provided by the kit (catalog #K752, BioVision, Inc.). Blood serum samples were used from 6 animals per treatment group in triplicate.

### Measurement of serum HDL and LDL cholesterol levels

Blood serum high-density lipoprotein (HDL) and low-density lipoprotein (LDL) cholesterols were measured using a HDL and LDL/VLDL cholesterols Quantification Colorimetric/Fluorometric Kit (BioVision, CA, USA). The colorimetric assay was used for the assay. Blood serum samples were used from 5 animals per treatment group in triplicate.

### Measurement of lipid accumulation and steatosis in liver

Liver lipid accumulation was visualized and measured using oil red O staining and the ImageJ 1.45s software. Liver tissue for oil red O staining were sectioned at 8 μm thickness using a cryostat (Leica CM 1850) at −25 °C. Cryosections were air dried for 10 minutes and then fixed in 10% formalin solution. Slides were then rinsed with running tap water and washed with 60% isopropanol. Sections were then stained with freshly prepared oil red O staining solution (3 g/L) (Sigma-Aldrich) for 15 min and then rinsed with 60% isopropanol and mounted with aqueous mountant (Sigma-Aldrich). 30 randomly selected lipid droplets in every treated mouse liver was measured for the quantification of lipid accumulation. Steatosis was assessed using H&E staining, as previously described^60^. 10 different regions of each mouse liver was measured to quantify hepatic steatosis.

### Measurement of apoptosis in tissues

Apoptosis assay was measured using the TACS^®^ 2 TdT-DAB *In Situ* Apoptosis Detection Kit (Trevigen, MD, USA), following the manufacturer’s protocol. Visualization of apoptotic nuclei in the heart, kidney and liver tissues was carried out at a final magnification of ×400, using tissues from 5 mice per treatment group. To count apoptotic nuclei, 9 fields of view were randomly selected from 3 stained sections from each mouse.

### Measurement of adipocyte size

Adipose tissue sections were stained with H&E. The size distribution of adipocytes in adipose tissue was measured using Image J 1.48v software, following the method previously described^25^. Adipose tissue from 8 mice per treatment group was stained and 40 randomly selected adipocytes were measured from each mouse.

### Measurement of tissue fibrosis

For assessment of fibrosis, tissue sections were stained with the Trichrome Stain (Masson) Kit (Sigma-Aldrich, MO, USA). Myocardial interstitial fibrosis, kidney fibrosis and liver fibrosis were quantified by measuring the blue stained area using Image J 1.48v software, as previously described^61^. Adipose tissue fibrosis was assessed by quantifying the blue stained area around the perimeter of adipocytes, as previously described^62^. The percentage fibrosis was calculated as the percentage of blue staining within the whole tissue section, as calculated using the ImageJ software. 10 mice/treatment group were used for the liver fibrosis analysis. 5 mice/treatment group were used for the cardiac, adipose, and kidney tissue fibrosis analysis. 3 stained sections from each animal was used to assess fibrosis.

### Assessment of inflammatory responses in macrophages

RAW 264.7 macrophages were seeded in 6 well plates at a density of 2.5 × 10^5^ cells/well. 12 h later, macrophages were treated with compound of interest for 16 h. The macrophages were then treated with or without 100 ng/mL LPS for 3 h and cells were harvested to isolate RNA for gene expression analysis. The assay was repeated two further times to obtain triplicate samples for quantitative real time PCR analysis.

### Statistical analysis

The Student’s *t* test (Microsoft Excel 2013) or 1-way-ANOVA with Dunnett’s correction as the Post-test analysis (Graphad Prism version 6) was used for comparison between experimental groups, as indicated in the manuscript figure legends. *p*-values of <0.05 were considered significant. Unless otherwise stated, all results are the average of three independent experiments and error bars are standard deviation.

## Additional Information

**How to cite this article:** Cho, H. *et al*. ENOblock, a unique small molecule inhibitor of the non-glycolytic functions of enolase, alleviates the symptoms of type 2 diabetes. *Sci. Rep.* **7**, 44186; doi: 10.1038/srep44186 (2017).

**Publisher's note:** Springer Nature remains neutral with regard to jurisdictional claims in published maps and institutional affiliations.

## Supplementary Material

Supplementary Information

## Figures and Tables

**Table 1:** Summary of the effects of ENOblock in T2DM mice.

Organ/tissue	Effect of ENOblock treatment
**Blood**	1) Reduction of glucose and LDL cholesterol levels.
	2) HDL cholesterol level unaffected.
**Liver**	1) Reduction of fibrosis and apoptosis.
	2) Less lipid accumulation and steatosis compared to rosiglitazone.
	3) Reduced expression of inflammatory markers IL-6 and TNF-α and gluconeogenesis regulator Pck-1.
	4) Reduced expression of key lipid biosynthesis regulators Srebp-1a and -1c without activating LXR response genes Scap and Abcg5.
**Adipose tissue**	1) Less weight gain and gonadal adipose mass compared to rosiglitazone.
	2) Reduced adipocyte size.
	3) Reduced expression of inflammatory markers TNF-α, CD11c and Mcp-1.
	4) Reduced expression of adipogenesis regulator PPARg and fibrosis marker collagen VI
**Heart**	1) Reduction of cardiac hypertrophy and cardiomyocyte width
	2) Reduced fibrosis and apoptosis.
	3) Increased expression of cardioprotective transcription factor Gata-4 and target genes α-MHC and Irap.
	4) Reduced expression of cardiac pathophysiology-related genes Kcnk1, Asah2, B4glant and MMP-3.
**Kidney**	1) Reduced fibrosis and apoptosis.
	2) Reduced expression of gluconeogenesis regulator Pck-1 and apoptosis-related genes angiotensin, Bax and p53.
	3) Increased expression of collagens 4a1 and 4a2; reduced expression of pro-fibrosis marker collagen 4a3.
