# Supplementary material for: ENOblock, a unique small molecule inhibitor of the non-glycolytic functions of enolase, alleviates the symptoms of type 2 diabetes
Source: Sci Rep. 2017 Mar 8;7:44186. doi: 10.1038/srep44186 (PMC5341156; doi:10.1038/srep44186)
Supplement: Supplementary Information [file srep44186-s1.doc]

**Supplementary information for the manuscript: ‘ENOblock, a unique small molecule inhibitor of the non-glycolytic functions of enolase, alleviates the symptoms of type 2 diabetes’**

**Haaglim Cho, JungIn Um, Ji-Hyung Lee, Woong-Hee Kim, Wan Seok Kang, So Hun Kim, Hyung-Ho Ha, Yong Chul Kim, Young-Keun Ahn, Da-Woon Jung and Darren R. Williams**

**Supplementary Figure 1)** Western blot to show nuclear localization of enolase in Huh7 hepatocytes treated with 10 µM ENOblock or 2 mM NaF (an enolase catalytic site inhibitor) for 48 h. α-tubulin and laminB were used to indicate the cytoplasmic and nuclear protein fractions, respectively. The band intensity of enolase relative to α-tubulin, for the cytoplasmic fraction, or laminB, for the nuclear fraction, is shown underneath (one representative western of three experimental repeats is shown).


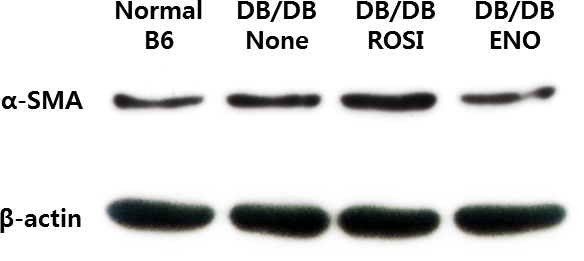


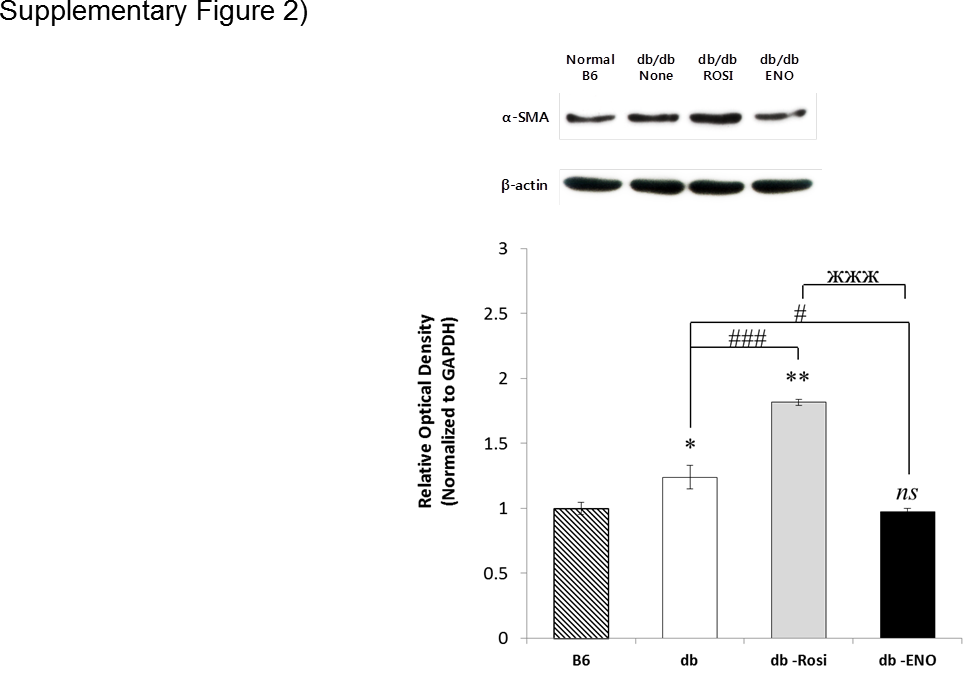


**Supplementary Figure 2)** Western blot analysis of α-SMA expression in the liver of db/db mice treatment with 8 mg/kg ENOblock or 8 mg/kg rosiglitazone for 7 weeks. Liver tissue from age-matched B6 mice is included for comparison with the db/db mice. Values are presented as means ± SE. Statistical analysis was carried out with a one-way ANOVA test, followed by a Dunnett's mutiple comparisons test and unpaired *t*-test. *ns*: not significantly different. *, ** or ***: significantly different from the corresponding normal B6 respectively with *p* <0.05, *p*<0.01, *p*<0.001; #, ## or ###: significantly different from the corresponding db-control respectively with *p*<0.05, *p*<0.01 or *p*<0.001; жжж: significantly different from the corresponding db-Rosi respectively with *p*<0.05, *p*<0.01 or *p*<0.001.

**Supplementary Table 1:** Primer sequences used in this study

| ***Abcg5*** | **F** | CGTGGCGGACCAAATGA | NM_031884 |
| --- | --- | --- | --- |
|  | **R** | GCTCGCCACTGGAAATTCC |  |
| ***Angiotensin*** | **F** | AGTGGGAGAGGTTCTCAATAGCA | NM_007428 |
|  | **R** | GACGTGGTCGGCTGTTCCT |  |
| ***Asah2*** | **F** | GCAAAGCGAACCTTCTCCAC | NM_018830 |
|  | **R** | ACTGGTAACAAACAAGAGGGTGA |  |
| ***α-Enolase(eno1)*** | **F** | GCACCCTCTTTCCTTGCTTTG | NM_023119 |
|  | **R** | CCTGAGAATAGACATGGCGAATT |  |
| ***α-MHC*** | **F** | GGGAAGACTGTGAACACAAAACG | NM_001164171 |
|  | **R** | CTTGCTACGGTCCCCTATGG |  |
| ***Bax*** | **F** | GGCCTTTTTGCTACAGGGTTT | L22472 |
|  | **R** | GTGTCTCCCCAGCCATCCT |  |
| ***B4galnt1*** | **F** | CAGCCCAGTTCTGGATAAACTCA | NM_001244617 |
|  | **R** | AGCTCCGGCTGCTGTAAGTC |  |
| ***Cd11c*** | **F** | CTGGATAGCCTTTCTTCTGCTG | NM_021334 |
|  | **R** | GCACACTGTGTCCGAACTCA |  |
| ***Col4a1*** | **F** | CAGCTGCCTGCGTAAGTTCA | NM_009931 |
|  | **R** | GCAGACGTTGTTGATGTTGCA |  |
| ***Col4a2*** | **F** | CCCATCTGACATCACACTTGTTG | NM_009932 |
|  | **R** | TGAGATTACGCCGGGTATCC |  |
| ***Col4a3*** | **F** | GGCAGAGCTCTCGAACCCTAT | NM_007734 |
|  | **R** | TGAACAGCTATGGCCATTGC |  |
| ***Col6a3*** | **F** | CATCGATGGATCCCGAAATG | NM_001243008 |
|  | **R** | CGGGTGGTGTCAAAGCCTAT |  |
| ***Cox-2*** | **F** | AGAGGTGTATCCCCCCACAGT | NM_011198 |
|  | **R** | TGCTCCCGAAGCCAGATG |  |
| ***c-Myc*** | **F** | GTCTTTCCCTACCCGCTCAAC | NM_001177352 |
|  | **R** | GTGGAATCGGACGAGGTACAG |  |
| ***Erbb2*** | **F** | TGGATGATTGACTCCGAATGTC | NM_001003817 |
|  | **R** | TGCCATACGGGAGAATTCTGA |  |
| ***Gata4*** | **F** | CCTCCCGCACGATTTCTG | NM_008092 |
|  | **R** | CTCAGGAAAAAGAAAATCCCAAATT |  |
| ***Glut-4*** | **F** | CATGGCTGTCGCTGGTTTC | AB008453 |
|  | **R** | AAACCCATGCCGACAATGA |  |
| ***Insig-1*** | **F** | CACCTGGGAGAACCACACAAG | NM_153526 |
|  | **R** | CACGGCAATACAGCGCATAA |  |
| ***Insig-2*** | **F** | ATGTGATCACGAGCATCTTTTCA | NM_001271531 |
|  | **R** | GGCTGTGCCGCAGCAT |  |
| ***Insig-2a*** | **F** | TCTGGTAGGTCCCACGTTCAG | AY156084 |
|  | **R** | GAACTGTGAAGTGAAGCAGACCAA |  |
| ***Insig-2b*** | **F** | TCTGGTAGGTCCCACGTTCAG | AY156085 |
|  | **R** | GAACTGTGAAGTGAAGCAGACCAA |  |
| ***Interleukin-6*** | **F** | TAGTCCTTCCTACCCCAATTTCC | NM_000600 |
|  | **R** | TTGGTCCTTAGCCACTCCTTC |  |
| ***Irap*** | **F** | TGCCATTATTCCTCTATGCTATGAACT | NM_172827 |
|  | **R** | CAGATCCCCTGAATGTCATTGA |  |
| ***Kcnk1*** | **F** | CAACGGTGTAGGACCAGACAAC | NM_008430 |
|  | **R** | CCAGTCCAAATAGATTCACCTTCTC |  |
| ***Mmp3*** | **F** | TCCTGATGTTGGTGGCTTCAG | NM_002422 |
|  | **R** | TGTCTTGGCAAATCCGGTGTA |  |
| ***Mcp-1(=ccl2)*** | **F** | CTCCTGCTCATAGCTACCACCAT | NM_011331 |
|  | **R** | GGGTGCTCACCGCATCTG |  |
| ***Pck-1*** | **F** | CTGCATAACGGTCTGGACTTC | NM_011044 |
|  | **R** | CAGCAACTGCCCGTACTCC |  |
| ***Pck-2*** | **F** | CCACAGGACTCCCCATGCT | NM_028994 |
|  | **R** | ATGGCTGCTATGTACCTCCC |  |
| ***p53*** | **F** | TGCATGGACGATCTGTTGCT | AB020317 |
|  | **R** | TTCACTTGGGCCTTCAAAAAA |  |
| ***Ppar-γ*** | **F** | GCCCACCAACTTCGGAATC | NM_001127330 |
|  | **R** | TGCGAGTGGTCTTCCATCAC |  |
| ***Srebp-1a*** | **F** | GATGTGCGAACTGGACACAG | NM_011480 |
|  | **R** | CATAGGGGGCGTCAAACAG |  |
| ***Srebp-1c*** | **F** | CCAGAGGGTGAGCCTGACAA | NM_001313979 |
|  | **R** | AGCCTCTGCAATTTCCAGATCT |  |
| ***Scap*** | **F** | TCTGACTTCTTCCTCCAGATGCT | NM_001001144 |
|  | **R** | CATCCGGCGAATGTCGAT |  |
| ***Tlr-4*** | **F** | CCTGGCTGGTTTACACGTC | NM_138557 |
|  | **R** | GACATTGCAGAAACATTCGC |  |
| ***Tnf-α*** | **F** | AAGCCTGTAGCCCACGTCGTA | NM_001278601 |
|  | **R** | GGCACCACTAGTTGGTTGTCTTTG |  |
